# Health Literacy but Not Frailty Predict Self-Care Behaviors in Patients with Heart Failure

**DOI:** 10.3390/ijerph15112474

**Published:** 2018-11-06

**Authors:** Youn-Jung Son, Dae Keun Shim, Eun Koung Seo, Eun Ji Seo

**Affiliations:** 1 Red Cross College of Nursing, Chung-Ang University, Seoul 06974, Korea; yjson@cau.ac.kr; 2 Medical director of Cardio-cerebrovascular Center, Good Morning Hospital, Pyeongtaek 17874, Korea; shimdk7626@gmail.com; 3 Director, Department of Nursing, Good Morning Hospital, Pyeongtaek 17874, Korea; sek2222@naver.com; 4 Ajou University College of Nursing and Research Institute of Nursing Science, Suwon 16499, Korea

**Keywords:** heart failure, health literacy, self-care, frailty

## Abstract

Heart failure (HF) is a chronic condition requiring continuous self-care. Health literacy is increasingly recognized as a key factor of self-care behaviors in patients with chronic diseases. Recently, frailty in chronic diseases has also been associated with self-care behaviors. However, relationships among health literacy, frailty, and self-care in the HF population are not well understood. Therefore, this cross-sectional study aimed to identify the impact of health literacy and frailty on self-care behaviors in patients with HF. Data were collected from 281 adults attending a cardiovascular outpatient clinic in Korea. Health literacy, frailty, and self-care behaviors were measured using Korean-validated instruments. The mean scores of health literacy and self-care behaviors were 8.89 (±3.44) and 31.49 (±5.38), respectively. The prevalence of frailty was around 26.3%. Health literacy was significantly associated with frailty and self-care behaviors. In a hierarchical linear regression analysis, health literacy was a significant determinant of self-care behaviors after adjusting for confounding variables, but frailty was not. Educational level was also a significant predictor of self-care behaviors. Our main findings showed that health literacy can facilitate improvements in HF self-care behaviors. Healthcare professionals should assess patients’ health literacy and educational backgrounds when designing self-management programs.

## 1. Introduction

Heart failure is the leading cause of mortality and healthcare costs worldwide [1]. It is a chronic progressive, irrevocable disease characterized by a heart’s functional or structural disorder that renders it unable to pump sufficient blood and oxygen [2]. Even though various treatment approaches are available, deteriorating heart failure symptoms and declining quality of life are serious health issues [3,4,5]. An aging population and improved medical technologies have increased the incidence of heart failure [1,2]. As heart failure prevalence continues to rise in Korea, it is critical to establish ways to prevent and manage it [6]. Important management strategies to reduce health issues in patients with heart failure include self-care behaviors like regular exercise, a balanced diet, sodium control, and weight control [2,7,8,9]. Therefore, studies on patients with heart failure have focused on interventions for the patients and their families to improve self-care. However, despite the positive health outcomes of self-care, many patients with heart failure still find it difficult to follow a self-care regimen [10].

Self-care is a cornerstone in long-term management of chronic heart failure [11]. A recent review reported that factors related to self-care behaviors in patients with heart failure include age, gender, educational level, symptom severity, frailty, depression, and health literacy [12]. However, the review did not reach a conclusion on the influence of patient-related variables like health literacy on self-care because they have not been sufficiently studied [12]. Another study also found that self-management strategies that did not consider the patients’ characteristics were ineffective [13]. This indicates that consideration of individual patient characteristics is important when designing self-management programs.

Knowing a patient’s level of comprehension of health information is particularly essential because health information allows patients to effectively apply treatment-related information to daily life. In this context, health literacy, which allows patients to assess and translate adequate health information to daily activities, is emerging as a facilitator of self-care behaviors for patients with heart failure [14,15]. Health literacy is the ability of individuals to obtain, read, understand, and use the basic healthcare information needed to make appropriate health decisions [16]. Health literacy plays a key role in applying information about disease and management to self-care. In particular, it is vital in filtering unnecessary or inaccurate information from various sources [17]. Patients with lower health literacy would not fully understand related health information despite an appropriate educational level and this may lead to misunderstanding of a disease and poor self-care [15]. However, the relationship between health literacy and heart failure self-care remains unclear [7] and needs further investigation in the heart failure population. A recent study reported that approximately 61% of Korean adults showed inadequate health literacy [18], while the prevalence of low health literacy in Korean patients with heart failure is not known.

Frailty is considered a biological syndrome associated with multisystem deterioration in physiological reserves [19]. It is reported to be predictive of major negative health-related outcomes including disability in the daily activities of patients with heart failure [20]. Thus, frailty in patients with heart failure may be negatively associated with self-care behaviors and, consequently, with the progress of the disease. A previous study on elderly patients with heart failure reported that the social components of frailty were adversely related to self-care ability [21]. Another prior study reported that the community-dwelling elderly with higher self-care ability were less frail [22]. However, the impact of frailty on self-care behaviors in patients with heart failure has yet to be fully understood.

Therefore, the relationships among health literacy, frailty, and self-care in patients with heart failure should be clearly identified. This study aimed to investigate whether health literacy and frailty are determinants of self-care behaviors in patients with heart failure after adjusting for the patients’ socio-demographic and clinical factors.

## 2. Materials and Methods

### 2.1. Study Design and Participants

This study adopted a cross-sectional research design. A convenience sample of patients with heart failure was recruited from the outpatient clinic of a general hospital in Pyeongtaek, South Korea. Convenience sampling in this study was chosen on the basis of availability, which was easily accessible within the defined study time period. From April to July 2018, a total of 281 patients who visited the outpatient clinic for post-discharge follow-up were recruited for this study.

The inclusion criteria were (1) aged 20 years or older; (2) articulate in the Korean language; (3) diagnosed with heart failure by a cardiologist based on clinical history, echocardiographic findings, and the presence of symptoms and diagnoses for at least one year; and (4) willing to participate. The exclusion criteria were (1) previously diagnosed with dementia or major depressive disorder, (2) presence of paralysis or physical impairment, (3) a life expectancy of less than 6 months, and (4) categorized as New York Heart Association (NYHA) class IV, requiring hospice or palliative care.

To determine the appropriate sample size for this study, a power analysis was conducted using G-power, version 3.1 [23]. Based on an alpha of 0.05, a power level of 0.95, and 10 predictors, the calculation found that 172 participants were needed for a moderate effect size of 0.15 [24]. Among the 290 patients initially recruited, nine patients were excluded for having incomplete questionnaires. Consequently, our final data analysis included a total of 281 patients, which met the criteria for a sufficient sample size.

### 2.2. Measurements

#### 2.2.1. Socio-Demographic and Clinical Characteristics

Socio-demographic data (age, gender, educational level, marital status, employment status, and monthly income) were obtained from standardized questions. The clinical information obtained from medical records included body mass index, duration of heart failure diagnosis, NYHA functional classification, physician-diagnosed comorbidities (hypertension, diabetes, cancer, heart attack, angina, asthma, chronic lung disease, arthritis, stroke, and chronic renal failure), prescribed medication, and ejection fraction (EF, %).

#### 2.2.2. Health Literacy

Health literacy was assessed using a 3-item set of brief screening questions developed by Chew, Bradley, and Boyko [25]. This scale assessed an individual’s ability to obtain and use health information to make appropriate health and medical care decisions. Patients were asked to answer the questions on a 5-point Likert scale (0–4). The total score was calculated by summing the three items and ranged from 0 to 12. Higher scores indicated more adequate health literacy. According to this scale, the patients with a total score of 11–12 were classified as having an adequate level of health literacy, a score of 7–10 was a marginal level, and a total score <6 was an inadequate level [25]. The Cronbach’s alpha from prior studies using this instrument was 0.82 [26]. In the present study, the scale’s Cronbach’s alpha was 0.88.

#### 2.2.3. Frailty

To assess the status of frailty, we adopted the Korean version of the FRAIL scale (K-FRAIL) [27]. The K-FRAIL scale, based on the original FRAIL scale (Fatigue, Resistance, Ambulation, Illness, and Loss of weight) [28], was determined to have clinical validity [27]. This self-reported scale had five items that assessed tiredness level, ambulation level, number of illnesses, and loss of weight. The total score was calculated by summing after a conversion to 0 or 1 and ranged from 0 to 5. According to this scale, the patients in this study with a total score of 0 were classified as “robust”, a total score of 1–2 was “pre-frail”, and a total score ≥3 was “frail” [27].

#### 2.2.4. Self-Care Behaviors

We assessed participants’ self-care behaviors with the Korean version of the European Heart Failure Self-Care Behavior Scale 9 items (EHFScBS-9) [29] which was originally developed for patients with heart failure by Jaarsma et al. [30]. The psychometric properties of the EHFScB-9 are reportedly reliable for the total scale and the consultation behavior subscale [30]. This self-reported scale had nine items that were rated on a 5-point Likert scale, from one (“I do not agree at all”) to five (“I completely agree”). The total score was calculated by summing the nine items and ranged from 9 to 45. Higher scores indicated better self-care behaviors. The reliability of the original scale ranged from a Cronbach’s alpha of 0.68 to 0.87 [30]. The Cronbach’s alpha for the present study was 0.70.

### 2.3. Data Collection and Ethical Considerations

This study protocol was reviewed and approved by the Institutional Review Boards of the authors’ institution (SBR-SUR-17-299). All patients were invited to participate in the study through telephone recruitment before visiting the outpatient clinic for follow-ups. A researcher and a trained research assistant confirmed patients as being eligible on the telephone recruitment list. If they were eligible, the researcher explained the study and the steps of agreement in detail. Written informed consent was obtained from patients who agreed to participate in the study at the outpatient clinic visit after fully understanding the study purpose and the rights and responsibilities of participants. The survey took approximately 15 min to complete. The consent information included details about the study aim, the confidentiality and anonymity of patient information, and the voluntary nature of participation.

### 2.4. Data Analysis

The collected data were analyzed using IBM SPSS Statistics version 23 (SPSS Inc., Chicago, IL, USA). Descriptive statistics were used to summarize data for presenting the patients’ characteristics. Continuous data were reported as means with standard deviations. Categorical data were expressed as numbers with percentages. The independent *t*-test, one-way analysis of variance, and the post hoc test (Scheffe test) were performed to identify differences between variables by characteristics. The correlations among variables were analyzed using Pearson’s correlation coefficient. Hierarchical regression was used to identify the impact of health literacy and frailty on self-care behaviors after adjusting for patient characteristics. All tests were performed at a *p* = 0.05 statistical significance level.

## 3. Results

### 3.1. Socio-Demographic and Clinical Characteristics

Most participants (60.9%, *n* = 171) were men, and the mean age was 68.7 (±11.1) with 37.4% (*n* = 108) of participants aged less than 60. Of the 281 patients, only 38.1% (*n* = 107) were well educated (above high school level), and 20.3% (*n* = 57) lived alone. The majority were unemployed (67.3%, *n* = 189) and had low monthly incomes (68.7%, *n* = 193) (Table 1).

In clinical characteristics, the majority belonged to NYHA class I (60.5%, *n* = 170), had a relatively short duration after heart failure diagnosis (1–2 years), and had a relatively normal LVEF (≥50%). The mean durations of heart failure and mean LVEF were 3.89 ± 2.89 years and 53.54 ± 11.39%, respectively (Table 2).

### 3.2. Differences in Health Literacy by Socio-Demographic and Clinical Characteristics

In Table 2, the health literacy level was higher in younger participants (F = 30.45, *p* < 0.001), men (*t* = 3.35, *p* < 0.001), and those with a higher educational level (F = 26.09, *p* < 0.001). Employed individuals (*t* = 7.66, *p* < 0.001) and those with higher monthly incomes (*t* = −8.30, *p* < 0.001) also had higher health literacy levels. In terms of clinical characteristics, the health literacy level was higher for NYHA I than for NYHA II and III (F = 19.34, *p* < 0.001). There were no differences in health literacy levels by heart failure duration, comorbidity, LVEF level, or medications except diuretics (*t* = −2.39, *p* = 0.019).

### 3.3. The Levels and Correlations among Health Literacy, Frailty, and Self-Care Behaviors

Table 3 shows the descriptive statistics and correlations by health literacy frailty and self-care behaviors in patients with heart failure. The health literacy and self-care mean scores were 8.89 (±3.44) and 31.49 (±5.38), respectively. The prevalence of low health literacy (inadequate and marginal levels) was 54.8% (*n* = 154). The prevalence of frailty was 26.3% (*n* = 74). Health literacy had a significantly negative correlation with frailty (r = −0.46, *p* < 0.001). In contrast, health literacy had a significantly positive correlation with self-care behaviors (*r* = 0.34, *p* < 0.001). Frailty had a significantly negative association with self-care behaviors (*r* = −0.32, *p* < 0.001).

### 3.4. Hierarchical Linear Regression Models for Determinants of Self-Care Behaviors

For the hierarchical regression analysis, socio-demographic (age, gender, educational level, job, and monthly income) and clinical variables (NYHA class and the status of taking diuretics) were first entered into Model 1 to determine the predictors of self-care behaviors, and then both health literacy and frailty were entered into Model 2 (Table 4).

In Model 1, younger individuals (*β* = −0.17, *p* = 0.017), those with a higher educational level (*β* = 0.23, *p* = 0.001), and those in the lower NYHA class (*β* = −0.16, *p* = 0.005) engaged more in self-care behaviors. In Model 2, health literacy (*β* = 0.14, *p* = 0.029) was a significant determinant of self-care behaviors after adjusting for socio-demographic and clinical variables, but frailty (*β* = −0.05, *p* = 0.441) was not. Educational level (*β* = 0.20, *p* = 0.005) remained significant after adding health literacy and frailty.

## 4. Discussion

The importance of self-care in patients with heart failure is well documented [10,11]. In recent years, some papers reported that health literacy and frailty were linked with self-care behaviors in patients with chronic diseases [15,19,20]. However, there is still a lack of information regarding the relationships among health literacy, frailty, and self-care behaviors in patients with heart failure [11]. Thus, the main purpose of the present study was to identify the impact of health literacy and frailty on self-care behaviors in patients with heart failure.

In this study, the mean health literacy score was 8.89, which was below the 11 to 12 score that indicated adequate health literacy [25]. It was also lower than the mean score of Korean patients undergoing percutaneous coronary intervention (10.72) [31] and the mean score of patients with diabetes (10.61) [32]. Moreover, in a previous study on patients with hypertension, the prevalence of low health literacy (marginal and inadequate levels) was 76.9% [26], while in this study on patients with heart failure, the prevalence was 54.8%. These findings suggest that health literacy might be associated with older age and medical conditions [2,4]. Namely, older patients who have limited physical function are likely to have lower health literacy resulting from cognitive decline and difficulty in using healthcare services [7,33]. In our univariate analysis, health literacy levels were higher in younger patients and in those with better physical functioning based on their NYHA classification. However, differences in cultural backgrounds, ethnicity, and chronic conditions may play a role. Thus, healthcare providers should carefully assess health literacy based on individual patient characteristics.

The average score of self-care behaviors in our study was 31.49, which was higher than the number reported in a previous review [12]. This study’s participants’ NYHA class was I to II, and LVEF was 40 or more in most cases, suggesting that heart failure severity was relatively low compared to the previous review [12]. The prevalence of frailty in the present study was about 26.3%. Another recent review reported an estimated overall prevalence of frailty in heart failure of around 44.5% [34]; however, the prevalence reported by individual papers in the review varied from 19% to 76% depending on patient characteristics. Accordingly, future studies are needed to explore the prevalence of frailty using a larger cohort sample.

A notable finding of this study was that health literacy had a significant impact on self-care behaviors after adjusting for patient characteristics and frailty. This finding was consistent with findings from previous studies [14,15,35]. Self-care behaviors included the process of planning and engaging in heath behavior changes independent of assistance from healthcare providers [11]. Thus, patients might need sufficient and reliable health information from diverse sources to prevent adverse health outcomes such as re-hospitalization resulting from symptom aggravation. According to previous studies [35,36,37], health literacy enables patients to collect and re-organize the information needed. Our finding solidified the evidence that health literacy can be an important factor for heart failure self-care behaviors. Thus, healthcare providers realizing the significance of health literacy can enhance self-care behaviors in combination with tailored education based on the health literacy level of patients with heart failure. Moreover, healthcare providers should be aware that early assessment for health literacy could be vital for delivering patient-focused education.

In addition, our study found that educational level was also a significant predictor of self-care behaviors. This result was in line with previous findings that educational level was related to health literacy [14,15] and self-care confidence [35]. In our study, patients who had higher educational levels were also likely to have higher levels of health literacy compared to those who had lower educational levels. This finding was also supported by a prior study that found that education was correlated with self-care behaviors [12]. Thus, this finding suggests that when designing patient education or self-management programs, it is necessary to consider the patients’ educational level. In other words, the patients’ misunderstanding in regard to health information can lead to non-compliance when they need to change lifestyles and make treatment-related decisions [38].

In this study’s univariate analysis, frailty was significantly associated with self-care behaviors even though frailty was not a significant predictor of self-care behaviors in the hierarchical linear regression. This finding might be related to the samples’ characteristics. Namely, the majority of the patients in our study belonged to NYHA class I with relatively normal EF and were therefore different from the frail heart failure populations of previous studies [39,40]. Frailty is also not a fixed condition that develops at one specific time point. Therefore, a prospective cohort study that identifies changes in frailty status in conjunction with heart failure progress may be a useful method.

There were several limitations to the present study. First, our study was not designed to evaluate longitudinal associations between health literacy, frailty, and self-care behaviors in patients with heart failure. The severity of frailty in the heart failure population may change over time, especially in elderly patients. Therefore, future studies are needed to determine the optimal screening intervals for these three factors to prevent adverse health outcomes including cognitive decline during the heart failure disease trajectory. Secondly, the study participants were limited to outpatients from a single tertiary care referral center using a convenience sampling; therefore, the results might not generalize to other clinical settings. Third, the present study included a small portion of the NYHA functional classes III and IV. The higher NYHA classes were positively associated with cognitive dysfunction in patients with heart failure. Finally, health literacy, frailty, and self-care behaviors in this study were self-reported measures which could lead to bias and overestimation of these variables.

## 5. Conclusions

The most critical components of managing heart failure are to plan optimal self-care interventions that consider the patients’ characteristics, share the plan with patients, and ultimately support them in performing adequate self-care. Our findings highlighted that high or adequate health literacy can lead to better compliance of self-care behaviors in patients with heart failure. Therefore, we recommend assessing health literacy in patients with heart failure using a screening tool. The purposes of the assessment are the monitoring of the level of health literacy and the identification of data for empirical and individual intervention. Through this, healthcare providers should be aware of the importance of early assessment for health literacy before patient education. Finally, offering tailored education based on the level of health literacy and educational background may help patients with heart failure improve self-care behaviors.

## Figures and Tables

**Table 1:** Patients’ socio-demographic characteristics and differences in health literacy (*n* = 281).

Characteristics	*n* (%)	Health Literacy		
		Mean ± SD	*t*/F	*p*
Age (years) *				
<60 ^a^	108 (37.4)	10.81 ± 2.24	30.45	<0.001
60–69 ^b^	100 (34.6)	10.27 ± 2.70		a,b > c
≥70 ^c^	81 (28.0)	7.58 ± 3.54		
Gender				
Men	171 (60.9)	9.44 ± 3.15	3.35	<0.001
Women	110 (39.1)	8.05 ± 3.71		
Educational level *				
Below elementary school ^a^	110 (39.1)	7.24 ± 3.42	26.09	<0.001
Middle school ^b^	64 (22.8)	9.41 ± 3.21		a < b,c
Above high school ^c^	107 (38.1)	10.29 ± 2.85		
Living arrangement				
Alone	57 (20.3)	8.33 ± 3.25	1.05	0.353
With spouse only	136 (48.4)	9.12 ± 3.38		
With family	88 (31.3)	8.90 ± 3.44		
Employment statue				
No	189 (67.3)	8.01 ± 3.54	7.66	<0.001
Yes	92 (32.7)	10.73 ± 2.35		
Monthly income (KRW)				
<1,000,000	193 (68.7)	8.01 ± 3.55	−8.30	<0.001
≥1,000,000	88 (31.3)	10.85 ± 2.14		

KRW = Korean Won. *—The same symbols indicate significant difference among groups (a, b and c) based on Scheffe test for multiple comparison.

**Table 2:** Patients’ clinical characteristics and differences in health literacy (*n* = 281).

Characteristics	*n* (%)	Health Literacy		
		Mean ± SD	*t* or F	*p*
NYHA class *				
I ^a^	170 (60.5)	9.86 ± 2.95	19.34	<0.001
II ^b^	86 (30.6)	7.29 ± 3.47		a > b,c
III ^c^	25 (8.9)	7.88 ± 3.86		
Duration of heart failure (years)				
1–2	140 (49.8)	8.97 ± 3.43	0.74	0.478
3–4	66 (23.5)	9.18 ± 3.41		
≥5	75 (26.7)	8.51 ± 3.50		
Comorbidity				
Hypertension, yes	128 (45.6)	9.16 ± 3.42	1.19	0.234
Diabetes, yes	92 (32.7)	8.96 ± 3.37	0.20	0.840
Coronary artery disease, yes	90 (32.0)	9.19 ± 3.47	0.97	0.330
Atrial fibrillation, yes	87 (31.0)	8.73 ± 3.52	−0.53	0.600
LVEF (%)				
≤40	42 (14.9)	9.02 ± 2.78	0.22	0.804
41–49	31 (11.0)	9.22 ± 3.48		
≥50	208 (74.0)	8.82 ± 3.56		
Prescribed medication				
ACEI, yes	20 (7.1)	9.50 ± 3.66	0.81	0.417
ARB, yes	119 (42.3)	8.94 ± 3.50	0.19	0.853
CCB, yes	16 (5.7)	8.13 ± 3.84	−0.92	0.357
Beta blocker, yes	173 (61.6)	8.92 ± 3.28	0.10	0.919
Diuretics, yes	101 (35.9)	8.24 ± 3.51	−2.39	0.019

ACEI = angiotensin-converting enzyme inhibitor; ARB = angiotensin receptor blocker; CCB = calcium channel blocker; LVEF = left ventricular ejection fraction; NYHA = New York Heart Association classification; SD = standard deviation. *—The same symbols indicate significant difference among groups (a, b and c) based on Scheffe test for multiple comparison.

**Table 3:** Descriptive statistics and correlations among health literacy, frailty, and self-care scores in patients with heart failure (HF) (*n* = 281).

Variables (Number of Items)	Min–Max	Mean ± SD or *n* (%)	Health Literacy	Frailty
			*r* (*p*)	*r* (*p*)
Self-care behaviors (9)	14–44	31.49 ± 5.38	0.34 (<0.001)	−0.32 (<0.001)
Health literacy (3)	0–12	8.89 ± 3.44		−0.46 (<0.001)
Adequate	11–12	127 (45.2)		
Marginal	7–10	81 (28.8)		
Inadequate	0–6	73 (26.0)		
Frailty (5)	0–4	1.65 ± 1.14		
Robust	0	43 (15.3)		
Pre-frail	1–2	164 (58.4)		
Frail	3–5	74 (26.3)		

SD = standard deviation.

**Table 4:** Hierarchical regression analysis for health literacy and frailty as determinants of self-care (*n* = 281).

Predictors	Model 1			Model 2		
	*β*	*t* (*p*)	95% CI	*β*	*t* (*p*)	95% CI
Age	−0.174	−2.407 (0.017)	−0.155 to −0.015	−0.140	−1.888 (0.060)	−0.139 to 0.003
Gender (1 = men)	0.014	1.705 (0.089)	−0.177 to 2.464	0.112	1.844 (0.066)	−0.083 to 2.539
Educational level (1 = above middle school)	0.226	3.271 (0.001)	0.551 to 2.217	0.195	2.805 (0.005)	0.356 to 2.034
Employment status (1 = yes)	0.176	1.165 (0.245)	−1.395 to 5.436	0.185	1.233 (0.219)	−1.267 to 5.509
Monthly income (1 = ≥1,000,000 KRW)	0.279	1.779 (0.076)	−0.344 to 6.808	0.265	1.705 (0.089)	−0.476 to 6.629
NYHA (1 = II − III)	−0.160	−2.839 (0.005)	−2.231 to −0.404	−0.112	−1.809 (0.072)	−1.925 to 0.081
Diuretics (1 = no)	0.013	0.236 (0.814)	−1.088 to 1.384	0.012	0.210 (0.834)	−1.096 to 1.357
Health literacy				0.140	2.191 (0.029)	0.022 to 0.417
Frailty				−0.051	−0.772 (0.441)	−2.198 to 0.960
*R*^2^, F(*p*), ∆*R*^2^	0.469, 10.987 (<0.001), 220			0.488, 9.433 (<0.001), 019		

CI = Confidence Interval; KRW = Korean Won; NYHA = New York Heart Association classification.
